# The impact of antenatal care on neonatal mortality in sub-Saharan Africa: A systematic review and meta-analysis

**DOI:** 10.1371/journal.pone.0222566

**Published:** 2019-09-13

**Authors:** Tesfalidet Tekelab, Catherine Chojenta, Roger Smith, Deborah Loxton

**Affiliations:** 1 Research Centre for Generational Health and Ageing, Faculty of Health and Medicine, University of Newcastle, Newcastle, New South Wales, Australia; 2 College of Medical and Health sciences, Wollega University, Nekemte, Oromia, Ethiopia; 3 The Mothers and Babies Research Centre at the Hunter Medical Research Institute, University of Newcastle, Newcastle, New South Wales, Australia; Centre Hospitalier Universitaire Vaudois, FRANCE

## Abstract

**Background:**

Newborns are at greatest risk of dying at and shortly after the time of birth. Newborn mortality remains an urgent concern and is an important indicator of child health, development and well-being. Studies examining the effectiveness of antenatal care on maternal and newborn health outcomes have provided conflicting results. The aim of this review and meta-analysis was to determine the pooled effect of antenatal care on neonatal mortality in sub-Saharan Africa.

**Methods:**

We searched PubMed, Medline, EMBASE, CINAHL and Google Scholar from September to November 2016 and then updated our search on April 13, 2019. Two independent reviewers extracted data from eligible studies. The quality of each included study was assessed using the Risk of Bias Assessment tool for Non-Randomized Studies (RoBANS). The results were reported based on risk ratio (RR) with 95% confidence intervals (CI) using a random-effects model.

**Results:**

Eight hundred and ninety eight studies were initially identified. During screening, 23 studies were found to be relevant for data extraction. Of these, only twelve studies fulfilled the inclusion criteria and were included in the analysis. In five of the twelve studies included in the analysis, antenatal care service utilization had a significant association with neonatal mortality. The pooled risk ratio by the random-effects model was 0.61 (95% CI: 0.43, 0.86) for neonates born to women who received at least one antenatal care visit by a skilled provider as compared to neonates born to women who did not receive antenatal care.

**Conclusion:**

This review indicates that utilization of at least one antenatal care visit by a skilled provider during pregnancy reduces the risk of neonatal mortality by 39% in sub-Saharan African countries. Thus, in order to accelerate progress towards the reduction of newborn deaths, all pregnant women should receive antenatal care during pregnancy.

## Introduction

Newborns are at greatest risk of dying at and shortly after the time of birth. Newborn mortality remains an urgent concern and is an important indicator of child health, development and well-being. Globally, child mortality is an unfinished agenda of the Millennium Development Goals (MDG4) and remains unacceptably high with approximately 11 deaths occurring every minute[1]. In 2015, there was progress in reducing child mortality by 3.9%. However, there has been slow progress in the reduction of neonatal mortality compared to post-neonatal mortality (the number of newborns dying between 28 and 364 days of age): 47% compared with 58% globally [1–4]. Neonatal mortality accounts for 44% of total under five deaths globally. For many newborns, their day of birth is also their day of death. On the day of birth almost one million neonatal deaths occur and close to two million neonates die in the first seven days of life [1, 5]. To achieve the Sustainable Development Strategies (SDS) by 2030, all countries have aimed to reduce neonatal mortality to 12 or fewer neonatal deaths per 1,000 live births and to ten or fewer by 2035. Therefore, newborn mortality has received greater attention in this post Millennium Development Goals era [6–8].

Even though under-five child mortality decreased in all regions of low-income countries, these countries are experiencing an increase in the proportion of newborn morbidity and death[1]. Every day in Africa, 3,100 newborns die within 28 days of birth. Studies report that in most countries in sub-Saharan Africa (SSA), the leading causes of newborn deaths were infections such as tetanus, sepsis and pneumonia, preterm birth complications and birth asphyxia **[**9–11]. SSA showed progress in the reduction of under-five mortality, by having a 4.1% annual rate of reduction between 2000 and 2016. However, neonatal mortality is still high [1, 4, 12]. This is thought to reflect, at least in part, non-use or underutilization of maternal health care services. About half of women and their babies in SSA do not receive skilled care during pregnancy, childbirth and the postnatal period [12, 13]. Effective and timely maternal health care before conception, as well as during pregnancy and childbirth, could save nearly 3 million newborns in high burden countries. Most neonatal deaths could be prevented by direct interventions [8, 14, 15]. Evidence suggests that two thirds of neonatal deaths could be prevented if all pregnant mothers and newborns had access to cost-effective and direct interventions as well as receiving care from skilled health care providers during pregnancy and childbirth [8, 16, 17].

Antenatal care (ANC) can be defined as the care provided by skilled health care providers to pregnant women and adolescent girls in order to ensure the best health conditions for both mother and baby during pregnancy [18]. Researchers examining the effectiveness of ANC interventions on maternal and newborn health outcomes have provided conflicting results [19–22]. In two recent systematic reviews conducted in both high- and low-income countries, it was shown that there is insufficient evidence of the effect of antenatal care on the reduction of newborn mortality [21, 23]. Both of these reviews, conducted only among socially disadvantaged and vulnerable women and compared lower number of ANC with the standard model (depending on the number of visits). However, in studies conducted in India and Indonesia, positive effects of ANC in preventing newborn deaths were indicated [24–26]. These studies used interventions such as supplementation of iron tablets during pregnancy. ANC interventions must be integral to any quality improvement in outcomes for both mothers and newborns. ANC addresses the most prevalent health issues that affect mothers and newborns [19–21, 27]. For instance, ANC helps health care providers to identify diseases such as hypertension, haemorrhage and diabetes mellitus, and treat conditions that could affect the wellbeing of the mother and baby.

In a recently published document, the World Health Organisation (WHO) now recommends a minimum of eight ANC visits to improve neonatal outcomes and to provide a more positive and women-centred experience for pregnant women [18]. It is estimated that ANC alone reduces neonatal mortality by 10–20% [20, 28], though the utilization of those services in SSA is inadequate [29]. Thus, considering the different and conflicting results concerning the effect of ANC on neonatal mortality in SSA [30–35], there is a need for assessing studies from SSA, where most maternal and newborn morbidity and mortality occur [10, 36]. A systematic review and meta-analysis is therefore necessary to critically evaluate relevant studies to provide up to date evidence about the effect of ANC on neonatal mortality. The aim of this systematic review and meta-analysis was to determine the pooled impact of ANC on neonatal mortality in sub-Saharan Africa.

## Methods

### Study selection

The Preferred Reporting Items for Systematic Reviews and Meta-Analyses (PRISMA) checklist was used in the formulation of the systematic review methodology [37]. The systematic review was registered on the PROSPERO prospective register of systematic reviews after piloting the study selection process (registration number PROSPERO 2016: CRD42016049143).

Studies were assessed for inclusion through a title and abstract review. Articles were selected from electronic database searches; a list of relevant studies was identified and the full text of the studies was obtained for further assessment. Reference lists of selected articles were assessed in order to identify other potential studies of interest.

### Search strategy

We searched the following databases from September to November 2016 and re-ran the search from March 23 to April 13, 2019 on PubMed, Medline, EMBASE, CINAHL and Google Scholar (S1 Table). We used the keywords “perinatal mortality”, “perinatal death”, “neonatal mortality”, “neonatal death”, “newborn mortality”, “newborn death”, “antenatal care”, “prenatal care”, “maternal health care”, “sub-Saharan Africa”, and “Africa south of the Sahara”. Grey literature was searched for on Google Scholar and ProQuest. While grey literature was searched, no studies included in the grey literature met the eligibility criteria of the current review.

### PICO criteria

#### Population

Live born neonates age less than 28 days.

#### Intervention

At least one antenatal care service utilization.

#### Comparison

Neonates born to women who received at least one antenatal care service as compared to neonates born to women who did not receive antenatal care.

#### Outcome

Newborn death during the first 28 days of life.

### Study inclusion criteria

#### Study design and language

All studies of any design were included if they met the following criteria: (i) the study involved a mother who gave birth without any specific risk factors; (ii) the study was conducted in the perinatal period (early neonatal mortality) if the authors defined a death during the first seven days of life and neonatal mortality if the authors defined a death during the first 28 days of life (iii); the study reported on the risk of perinatal or neonatal mortality; (iv) utilization of antenatal care was considered as a possible factor for neonatal mortality; and (v) the article was published in English.

#### Exclusion criteria

Studies that focused only on the number of ANC visits were excluded from the review. Studies were excluded on the basis of a full-text assessment and the main characteristics of these excluded studies are shown in S1 File.

### Data extraction

The relevance of the studies were checked based on their topic, aims and methodology. Two independent reviewers (TT & CC) completed the data extraction, using a standardized form with clear inclusion and exclusion criteria. Disagreement between the reviewers was resolved by discussion between the authors or by consulting the third author. For each study, we recorded the first author’s last name, year of publication, study setting, study design, study period, sample size, response rate, population, outcome definition, comparison groups and effect estimate. The outcome variables were perinatal and neonatal mortality. Neonatal mortality was defined as a death occurring during the first four weeks (28 days) after the birth of a live baby [38]. The exposure variable ANC was classified as ‘no ANC visit’ and ‘one or more ANC visits’. Where necessary, we contacted study authors to request further information, such as missing data.

### Quality assessment

All studies selected for inclusion in the review were assessed rigorously by review authors. The quality of each included study was assessed using the Risk of Bias Assessment tool for Non-Randomized Studies (RoBANS) [39]. Studies were assessed across six categories (selection bias, confounding bias, performance bias, detection bias, attrition bias and reporting bias). Each domain was allocated one of three possible categories for each of the included studies: ‘low risk’, ‘high risk’, and ‘unclear’. RoBANS is shown in S2 Table.

### Data synthesis and analysis

Statistical analysis was carried out in Stata 11. The overall effect of ANC on neonatal mortality was carried out by using a DerSimonian and Laird random effects model [40] and risk ratio was measured with 95% CI. To assess heterogeneity among studies, we calculated the I^2^ statistic, which describes the percentage of total variation among studies due to heterogeneity rather than to chance. An I^2^ statistical value of 30% to 60% may represent moderate heterogeneity, whereas 50% to 90% may represent substantial heterogeneity and 75% to 100% may represent considerable heterogeneity [41].

The ‘leave-one-out’ method was used to conduct a sensitivity analysis in order to evaluate the stability of the results and to test whether any single study had an influence on the meta-analysis. Moreover, subgroup analyses were conducted to explore possible sources of heterogeneity.

Potential publication bias was investigated by visual inspection of funnel plots and Egger’s linear regression test [42, 43].

### Operational definition

A woman who received ANC was defined as ‘a women having at least one health facility visit for a pregnancy check-up by skilled attendants during pregnancy’. For instance, the included studies defined ANC as:

- Having a health facility visit for a pregnancy check-up by skilled attendants during pregnancy [31];
- Care given during pregnancy by a skilled birth attendant [44, 45]; and
- At least one visit with a skilled birth attendant during pregnancy [32–34, 46].

#### Neonatal death

Death of a newborn during the first 28 days of life.

## Results

### Search results

**Fig 1:**
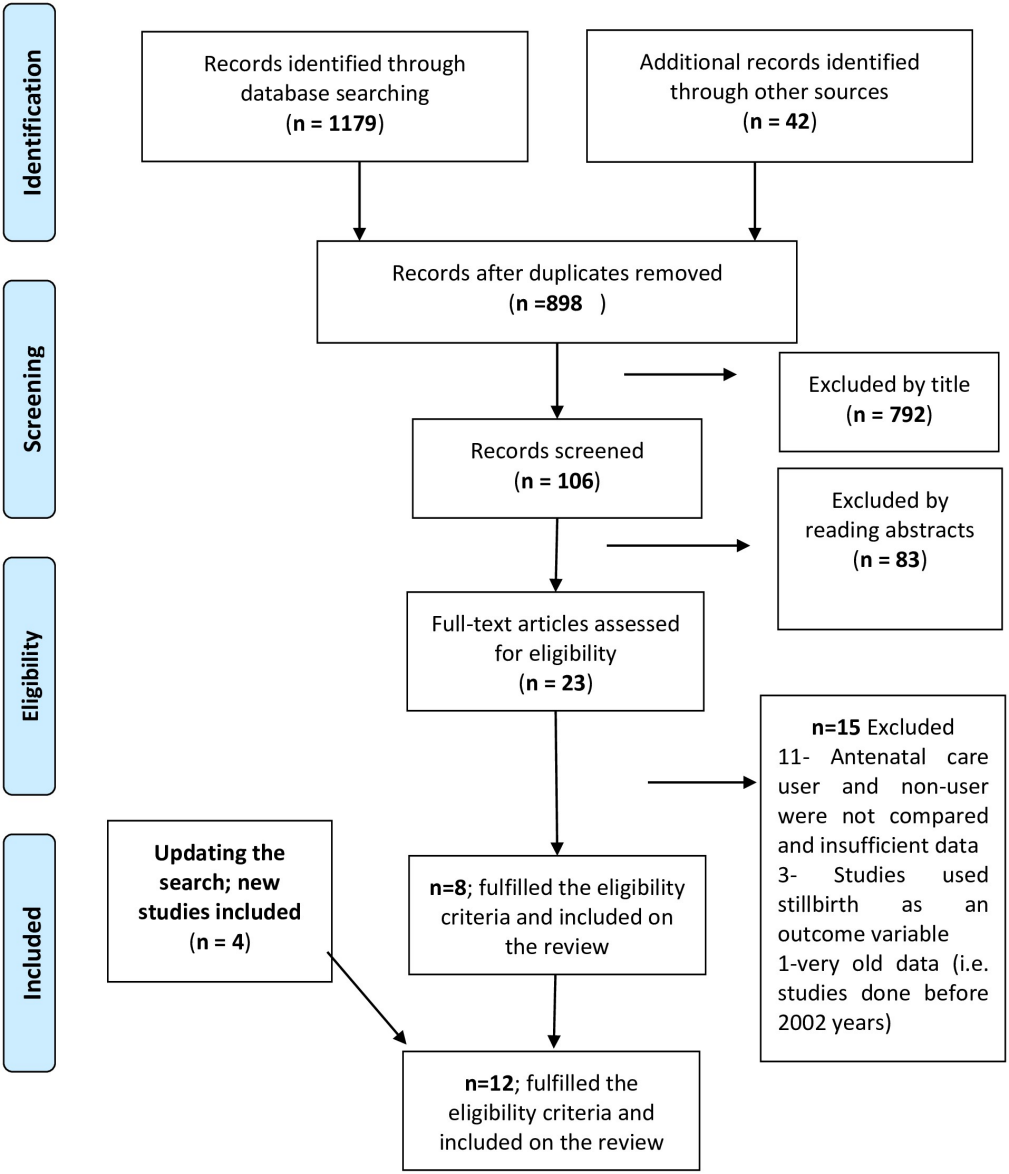
Flow chart showing identification, screening and inclusion of studies for systematic review and meta-analysis of the impact of antenatal care on neonatal mortality in sub-Saharan Africa.

As shown in Fig 1, our study search yielded 1,179 published articles on neonatal mortality. After removing duplicates, 898 records remained, of which 792 were excluded during the initial screening as their titles were not relevant. We then assessed the abstracts of 106 studies before excluding 83 more studies. Finally, after applying the inclusion and exclusion criteria, eight studies were included. Four studies were added after updating the search. Therefore, a total of twelve studies were included in the review.

### Study characteristics

The review included studies from seven countries. The design of the studies were five cohort studies, two case-control studies and five cross-sectional studies [31–35, 44–50]. The sample size ranged from 238 to 27,147. Three fourths of the studies were community-based studies and the remaining three were facility-based studies. The publication year of the included studies ranged from 2009 to 2019. A total of 79,990 live births were included, of which 2,107 died within 28 days of birth, making the weighted neonatal mortality rate 26.3 per 1,000 live births (Table 1).

**Table 1:** Characteristics of studies included in the systematic review and meta-analysis of the impact of antenatal care on neonatal mortality in sub-Saharan Africa.

First author & year of publication	Country	Study setting	Study design	Study period	Outcome definition	Antenatal care definition	Sample size	Received ANC		Not received ANC	
								Live births	Neonatal deaths	Live births	Neonatal deaths
Engmann C, 2009	Democratic Republic of Congo	Facility-based	Cross-sectional from RCT	June 2005 and January 2007	Death of a live born infant at or before 7 days of life	At least one visit with a skilled birth attendant during pregnancy	8257	7604	231	355	24
Diallo AH, 2011	Burkina Faso	Community-based	Prospective cohort study from RCT	June 2006 to May 2007	Death of any live born infant within 28 days of his/her birth	Use of health services during pregnancy[61]	895	626	28	238	12
Nankabirwa V, 2011	Uganda	Community-based	Prospective cohort study from RCT	January 2006 to May 2008	Number of newborn deaths in the first 7 days of life	Women who attended health facilities to receive care during pregnancy by a skilled birth attendant	835	600	12	235	6
Debelew GT, 2014	Ethiopia	Community-based	Prospective cohort study	September 2012 to December 2013	Death of an infant before 28 completed days	Having at least one health facility visit for a pregnancy check-up by skilled attendants during pregnancy	3604	2645	78	819	32
Welaga P, 2013	Ghana	Community-based	Cross-sectional	January 2003 to 2009	Not specified	Care during pregnancy with a skilled birth attendant [62]	18237	12460	301	5291	123
Kolola T, 2016	Ethiopia	Community-based	Case-control	January to May 2015	Deceased newborns within 28 days of birth	Health facility visits during pregnancy	336	313	72	23	12
Ezeh OK, 2014	Nigeria	Community-based	Cross-sectional	2003 and 2008	Death of a neonate between birth and 1 month of life	Care provided by a skilled health worker during pregnancy [63]	27147	9421	232	6316	169
Engmann C, 2012	Ghana	Community-based	Cross-sectional	January 2002 to December 2008	Death of a live born infant at or before 7 days of life	Care with a skilled birth attendant during pregnancy	20497	11939	179	5372	87
Arunda M, 2017	Kenya	Community-based	Cross-sectional	May 7 to October 20, 2014	Death of a baby before reaching 28 days (one month) of age	Mother visited a skilled provider for check-ups and pregnancy-related advice during pregnancy	14,190	13414	167	539	24
Orsido TT, 2019	Ethiopia	Facility-based	Retrospective cohort	October 2015 to October 2017	Death of a newborn within 28 days	Having at least one health facility visit for a pregnancy check-up by skilled birth attendant	964	719	79	86	80
Kidus F, 2019	Ethiopia	Community-based	Case-control	February 1 to December 30, 2013	Newborn death within 28 days	Pregnancy care received from skilled providers [64]	238	131	47	97	67
Farah AE, 2018	Ethiopia	Facility-based	Retrospective cohort	August 2014 to May 2017.	Newborn death within 28 days	Pregnancy care received from skilled providers [64]	792	666	37	81	8

### Risk of bias

The assessment of the risk of bias in all studies included are shown in Table 2. The risk of bias in the selection of participants was low in all studies, except in one study which is unclear. However, the risk of confounding bias was high in ten studies, but low in two studies which adjusted for major confounding variables during analysis. The risk of performance bias due to an inadequate measurement of risk factors was low in seven studies, which described exposure or risk factors clearly; it was unclear in three studies and high in two studies. The risk of detection bias due to an inadequate measurement of outcome was low in eight studies and high in the remaining studies. The risk of attrition bias due to an inadequate handling of incomplete outcome data was low in ten studies, unclear in one and high in one study. The risk of reporting bias was low in all studies due to selective reporting of the outcomes. See S2 Table.

**Table 2:** Risk of bias of included non-randomized studies on impact of antenatal care on neonatal mortality in sub-Saharan Africa.

Author/Year	Selection of participant (selection bias)	Confounding variables (confounding bias)	Measurement of exposure (performance bias)	Measurement of outcomes (detection bias)	Incomplete outcome data (attrition bias)	Selective outcome reporting (reporting bias)
Diallo AH, 2011	Low	High	Low	High	Unclear	Low
Engmann C, 2009	Low	High	Unclear	High	Low	Low
Nankabirwa V, 2011	Low	High	Low	High	Low	Low
Debelew GT, 2014	Low	Low	Low	Low	Low	Low
Kidus F, 2019	Low	Low	High	Low	Low	Low
Welaga P, 2013	Low	High	High	Low	Low	Low
Kolola T, 2016	Low	High	Low	High	Low	Low
Ezeh OK, 2014	Low	High	Low	Low	High	Low
Engmann C, 2012	Low	High	Unclear	Low	Low	Low
Arunda M, 2017	Unclear	High	Low	Low	Low	Low
Orsido TT, 2019	Low	High	Low	Low	Low	Low
Farah AE, 2018	Low	High	Unclear	Low	Low	Low

### Pooled effect size of antenatal care on neonatal mortality

**Fig 2:**
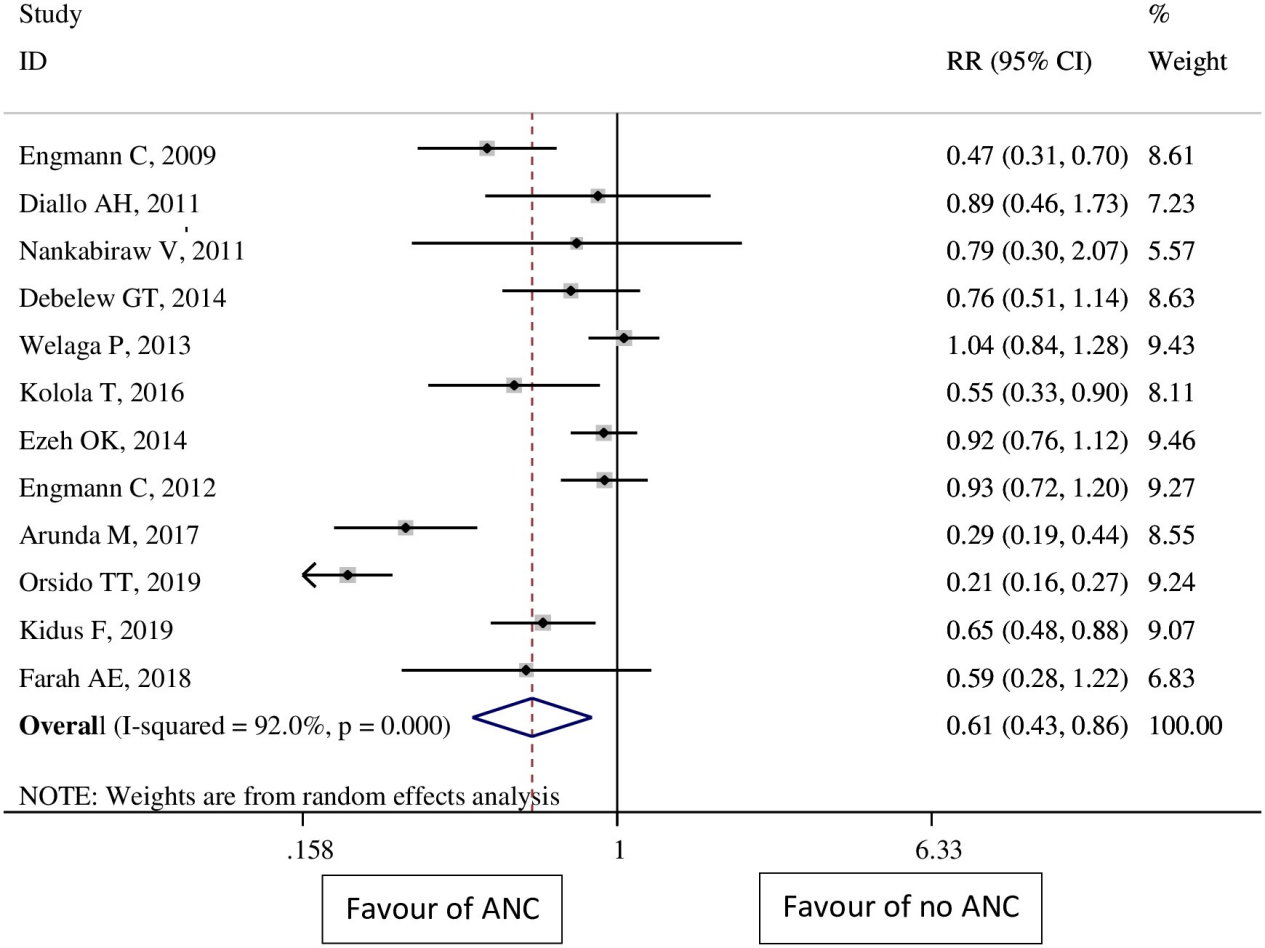
Overall pooled estimates of the impact of antenatal care on neonatal mortality in sub-Saharan Africa.

Among the twelve studies included in the analysis, five showed statistically significant associations between ANC services utilization and neonatal mortality, and seven showed non-significant effects. The pooled effect size by the random-effects model was 0.61 (95% CI: 0.43, 0.86) for neonates born to women who received at least one ANC visit as compared to neonates born to women who did not receive ANC (Fig 2).

### Sources of heterogeneity

There was substantial heterogeneity across studies overall (I^2^>92%, P value for heterogeneity <0.001), as well as within subgroups for study design, study region, sample size and study place. In a subgroup analysis, a significant reduction of neonatal mortality was observed in case-control studies (RR = 0.62 [95% CI 0.48–0.80]; P = 0.57 for the heterogeneity test, I^2^ = 0.0%,). When the included studies were stratified based on study region, the heterogeneity disappeared in the study done in West Africa (P for heterogeneity = 0.84, *I*^*2*^ = 0.0%); however, heterogeneity was still present in studies done in East Africa (P for heterogeneity<0.001, *I*^*2*^ = 88.5%; Table 3).

**Table 3:** Sub-group analysis of studies included in meta-analysis of impact of antenatal care on neonatal mortality in sub-Saharan Africa.

Sub-group	Random effects RR(95%CI)	I-squared, P-value
**Study design**		
Cohort	0.56(0.26–1.21)	91.1%, P<0.001
Case-control	0.62(0.48–0.80)	0.0%, P = 0.57
Cross-sectional	0.68(0.47–1.0)	89.8%, P< 0.001
**Study region**		
West Africa	0.96(0.85–1.09)	0.0%, P = 0.84
East Africa	0.48(0.30–0.77)	88.5%,P<0.001
Central Africa	0.47(0.31–0.70)	-
**Sample Size**		
<500	0.62(0.48–0.80)	0.0%, P = 0.57
500–1000	0.52(0.21–1.27)	88.9%, P<0.001
>1000	0.69(0.50–0.96)	87.2%,P<0.001
**Study place**		
Facility-based	0.36(0.18–0.73)	87.1%,P<0.001
Community-based	0.73(0.57–0.93)	78.3%,P<0.001

**Fig 3:**
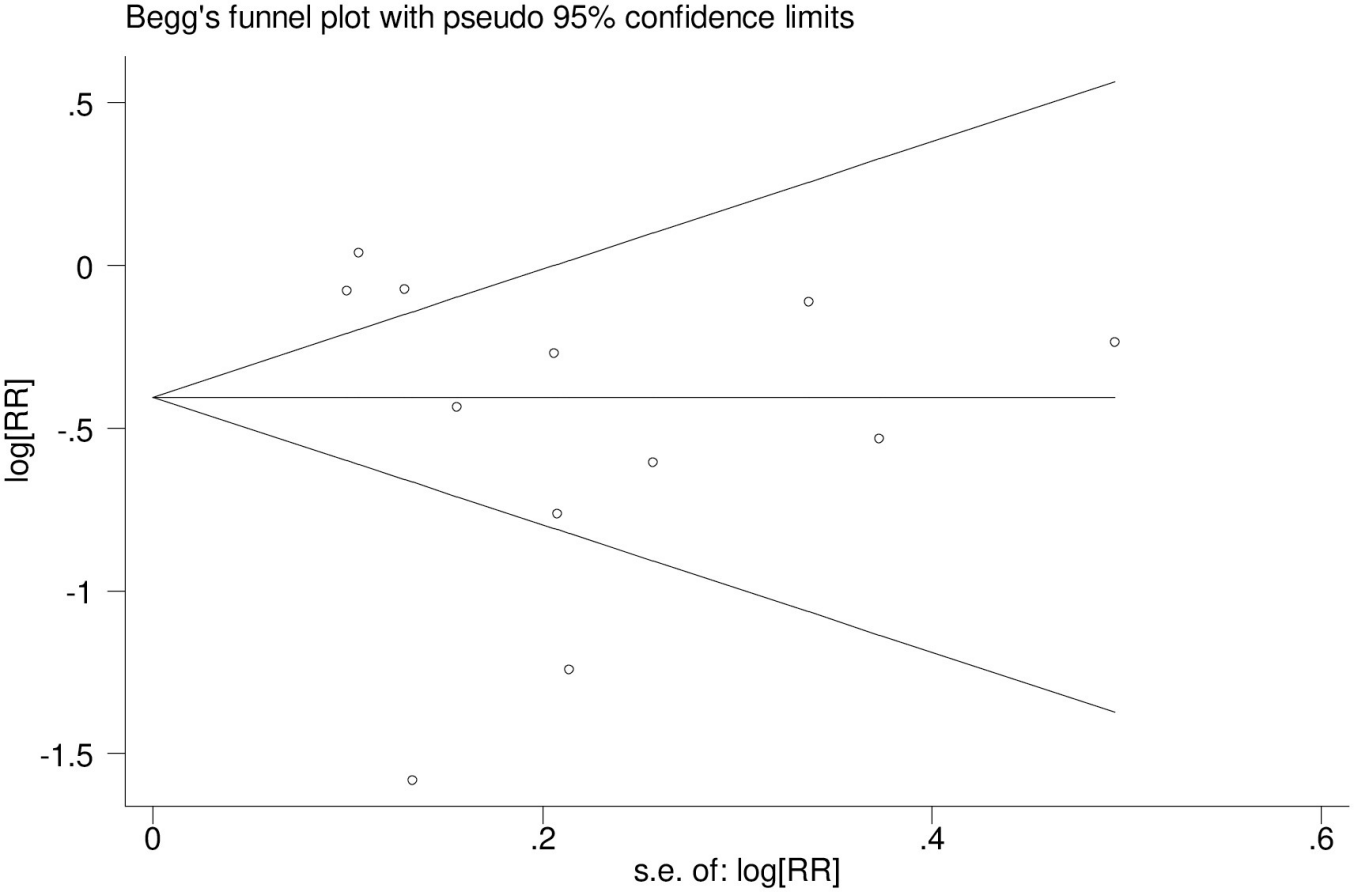
Funnel plot of the studies included in the meta-analysis of the impact of antenatal care on neonatal mortality in sub-Saharan Africa.

Visual observation of the funnel plot symmetry, the Egger test (P = 0.273) and Begg’s Test (P = 0.444) showed no publication bias (Fig 3). These findings indicate no major threat to the validity of the review.

## Discussion

This systematic review and meta-analysis assessed the pooled effect of ANC on neonatal mortality in SSA. The review is the first of its kind to be done in SSA and clearly shows the benefit to neonatal outcomes of ANC. Previous studies did not report the pooled effect of ANC on newborn health and focused only on the effect of health facility delivery on neonatal mortality [51, 52]. The current review provides important information to policy makers who are working in the area of maternal and infant health care in SSA by delivering robust evidence to evaluate policies related to ANC and newborn mortality. This review may help to strengthen ANC policies and programs, which may improve newborn health in SSA. Moreover, ANC will create an opportunity for women and their newborns to receive different interventions, including immunization and other necessary pregnancy related information on preventive measures that may have an impact on preventing newborn death.

Attendance of at least one ANC appointment had a statistically significant effect on neonatal mortality. We found a 39% lower risk of neonatal mortality among women who attended at least one ANC visit in SSA. All of the included studies in this review were from different parts of SSA. Therefore, the review provides the most comprehensive evidence for countries in SSA to promote the importance of ANC for the health of newborns. This finding is in line with other studies done in low- and middle-income countries [30, 51, 53, 54]. A survival analysis in low- and middle-income countries found a 55% lower risk of neonatal mortality among women who met the previous WHO recommendations of four ANC visits, and a 32% lower risk among those who had at least one ANC visit [30]. Similarly, in an analysis of the Indonesian Demographic and Health Survey, it was reported that use of postnatal care was not associated with neonatal survival, but ANC reduced the risk of neonatal death by 51% [53]. This may be due to the mother receiving important advice during the pregnancy and receiving iron, folic acid and tetanus immunizations, which also have a positive effect in reducing neonatal mortality. In addition, ANC has indirect benefits since women attending ANC are more likely to have their delivery assisted by a skilled birth attendant or give birth in health facilities [53].

According to a recent United Nations Children’s Fund (UNICEF) report, the majority of newborn deaths are easily preventable with simple, cost-effective interventions administered during pregnancy and childbirth [55]. However, there is a high degree of variability in the use and quality of maternal health care services provided to pregnant women and their newborns. Many women and newborns miss out on key interventions that can save their lives [55]. In previous literature, it has been indicated that causes of newborn deaths in Africa could be minimized or prevented through ANC interventions such as tetanus vaccination and iron supplementation provided during pregnancy [9, 56–59]. In this review, we found the most common causes of neonatal mortality were birth asphyxia, infection, preterm birth and birth injury. These findings are consistent with other studies conducted elsewhere [12, 60]. This may be due to non-use of maternal health care services during pregnancy and childbirth. The other reason may be a lack of counselling during pregnancy to prevent maternal and newborn complications and inadequate use of ANC interventions.

The findings of this systematic review and meta-analysis need careful consideration, bearing in mind its limitations. Firstly, the findings are based on the data extracted from observational studies that are associated with inherent biases. Secondly, the included studies were limited to English only, which may result in missing studies that could have been published in other languages. Thirdly, there was inconsistent classification of ANC visits in the included studies, so it was difficult to show the pooled effect size of each ANC visit (based on the number of visits). Fourthly, we are unable to show the impact of specific ANC interventions on neonatal mortality because there is insufficient information in relation to how ANC was defined and which specific interventions were performed in the included studies. This points to the need for more research in this area to establish which aspects of ANC are the most important for infant outcomes. Despite these limitations, we conducted a comprehensive search of databases to include all relevant studies, and sub-group analyses were conducted to determine whether any specific study-level factor explained the results.

## Conclusion

This review on the impact of ANC on neonatal mortality in SSA found that women who received one ANC visit by a skilled provider were less likely to experience neonatal mortality than those who did not. Thus, to accelerate progress towards the reduction of newborn deaths, all pregnant women should receive ANC by a skilled provider. Moreover, the health care provider should provide counselling to pregnant women during ANC visits to increase health facility delivery and this in turn may reduce neonatal mortality by preventing sepsis and by managing and treating preterm birth. Stakeholders should develop strategies to increase the utilization of ANC.

## Supporting information

S1 File Excluded articles and reason. (DOCX)

S2 File Completed PRISMA Checklist impact of ANC on neonatal mortality. (DOC)

S1 Table Search method used in Medline. (DOCX)

S2 Table The Risk of Bias Assessment tool for RoBANS. (DOCX)
