# Supplementary material for: The impact of antenatal care on neonatal mortality in sub-Saharan Africa: A systematic review and meta-analysis
Source: PLoS One. 2019 Sep 13;14(9):e0222566. doi: 10.1371/journal.pone.0222566 (PMC6743758; doi:10.1371/journal.pone.0222566)
Supplement: S1 File — (DOCX) [file pone.0222566.s001.docx]

**Excluded articles**

The following 11 articles were excluded after reviewing the full texts because of exposure and outcome definition , mixing of the outcome in the analysis part, consider risk group and insufficient data.

| S.No | Study | Reason for exclusion |
| --- | --- | --- |
| 1 | Feresu SA, Harlow SD, Welch K, Gillespie BW. Incidence of stillbirth and perinatal mortality and their associated factors among women delivering at Harare Maternity Hospital, Zimbabwe: a cross-sectional retrospective analysis. BMC pregnancy and childbirth. 2005 May 5;5(1):1. | The data collected before 2001 |
| 2 | Mekonnen Y, Tensou B, Telake DS, Degefie T, Bekele A. Neonatal mortality in Ethiopia: trends and determinants. BMC public health. 2013 May 17;13(1):1. | The analysed data include only antenatal care user |
| 3 | Bashir AO, Ibrahim GH, Bashier IA, Adam I. Neonatal mortality in Sudan: analysis of the Sudan household survey, 2010. BMC public health. 2013 Apr 1;13(1):1. | Define antenatal care different from our review aim.  Were constructed from a multiple 9 alternative health providers. Women who answered: Traditional birth attendant or Community health worker, no one was coded as non / traditional health providers (THP) users. whereas women who answered doctor or nurse or village midwife or health visitor or medical assistant were coded professional health providers (PHP) users. However, if women mentioned that they visited both health provider belongs to THP group and one belongs to PHP group, they were considered as PHP users. |
| 4 | Dahiru T. Surviving the First Day in Nigeria: Risk Factors and Protectors. American Journal of Public Health Research. 2015 Jul 1;3(4A):19-26. | The sample not written correctly |
| 5 | Yego F, D’Este C, Byles J, Nyongesa P, Williams JS. A case-control study of risk factors for fetal and early neonatal deaths in a tertiary hospital in Kenya. BMC pregnancy and childbirth. 2014 Nov 29;14(1):1. | The analysis was done by mixing the infant and newborn death |
| 6 | Mengesha HG, Wuneh AD, Lerebo WT, Tekle TH. Survival of neonates and predictors of their mortality in Tigray region, Northern Ethiopia: prospective cohort study. BMC Pregnancy and Childbirth. 2016 Aug 2;16(1):202. | Insufficient data |
| 7 | Adetola AO, Tongo OO, Orimadegun AE, Osinusi K. Neonatal mortality in an urban population in Ibadan, Nigeria. Pediatrics & Neonatology. 2011 Oct 31;52(5):243-50. | The exposures wrongly calculated |
| 8 | Kananura RM, Tetui M, Mutebi A, Bua JN, Waiswa P, Kiwanuka SN, Ekirapa-Kiracho E, Makumbi F. The neonatal mortality and its determinants in rural communities of Eastern Uganda. Reproductive health. 2016 Feb 16;13(1):1. | Compares antenatal care visit with neonatal mortality |
| 10 | Musooko M, Kakaire O, Nakimuli A, Nakubulwa S, Nankunda J, Osinde MO, Mbalinda SN, Kakande N, Kaye DK. Incidence and risk factors for early neonatal mortality in newborns with severe perinatal morbidity in Uganda. International Journal of Gynecology & Obstetrics. 2014 Nov 30;127(2):201-5. | Assessed based on risk factors |
| 11 | Akinyemi JO, Bamgboye EA, Ayeni O. Trends in neonatal mortality in Nigeria and effects of bio-demographic and maternal  Characteristics. BMC Pediatrics (2015) 15:36 | No comparative group. |
| 12 | Worku B, Kassie A, Mekasha A, Tilahun B, Worku A. Predictors of early neonatal mortality at a neonatal intensive care unit of a specialized referral teaching hospital in Ethiopia. Ethiopian Journal of Health Development. 2012 Jan 1;26(3):200-7. | The study included newborn death from the intensive neonatal care |
| 13 | Zwane E, Masango S. Factors influencing neonatal mortality: an analysis using the Swaziland Demographic Health Survey 2007. Journal of Public Health in Africa. 2012 Oct 22;3(2):18. | Not used antenatal care as exposure variables |
| 14 | Negera Wakgari, Eshetu Wencheko. Risk factors of neonatal mortality in Ethiopia.  *Ethiop. J. Health Dev.* 2013;27(3):192-199] | Unable to find the number of live birth |
| 15 | Kaboré R, Meda IB, Koulidiati JL, Millogo T, Kouanda S. Factors associated with very early neonatal mortality in Burkina Faso: A matched case–control study. International Journal of Gynecology & Obstetrics. 2016 Nov 1;135(S1). | Absence of comparative group |
