# Supplementary material for: The impact of antenatal care on neonatal mortality in sub-Saharan Africa: A systematic review and meta-analysis
Source: PLoS One. 2019 Sep 13;14(9):e0222566. doi: 10.1371/journal.pone.0222566 (PMC6743758; doi:10.1371/journal.pone.0222566)
Supplement: S1 Table — (DOCX) [file pone.0222566.s003.docx]

Database(s): **Ovid MEDLINE(R) and Epub Ahead of Print, In-Process & Other Non-Indexed Citations and Daily**1946 to May 28, 2019 
Search Strategy:

| **#** | **Searches** | **Results** |
| --- | --- | --- |
| 1 | "Sub-Saharan".mp. or exp "Africa South of the Sahara"/ | 203294 |
| 2 | Maternal Health Services/ or Prenatal Care/ or antenatal care.mp. | 41450 |
| 3 | perinatal.mp. or Perinatal Care/ | 70286 |
| 4 | 2 or 3 | 106782 |
| 5 | fetal mortality/ or infant mortality/ or perinatal mortality/ | 29442 |
| 6 | ((bab* or newborn* or neonat* or fetus* or Prenatal*) adj5 (death* or mortalit* or fatal* or outcom* or complicat*)).mp. [mp=title, abstract, original title, name of substance word, subject heading word, floating sub-heading word, keyword heading word, organism supplementary concept word, protocol supplementary concept word, rare disease supplementary concept word, unique identifier, synonyms] | 59660 |
| 7 | 5 or 6 | 81149 |
| 8 | 1 and 4 and 7 | 1727 |
| 9 | limit 8 to (english language ) | 1176 |
