# Supplementary material for: The impact of antenatal care on neonatal mortality in sub-Saharan Africa: A systematic review and meta-analysis
Source: PLoS One. 2019 Sep 13;14(9):e0222566. doi: 10.1371/journal.pone.0222566 (PMC6743758; doi:10.1371/journal.pone.0222566)
Supplement: S2 Table — (DOCX) [file pone.0222566.s004.docx]

***Risk of bias assessments (RoBANS)***

| **Study ID** | **Selection of participants(Selection bias)** | **Confounding variables(confounding bias)** | **Measurement of exposure(Performance bias)** | **Blinding of outcomes assessment(Detection bias)** | **Incomplete outcome data(Attrition bias)** | **Selective outcome reporting(Reporting bias)** |
| --- | --- | --- | --- | --- | --- | --- |
| Diallo AH,2011 | **Low.**  All pregnant women selected from the same village and no complication or neonatal death before the study. Women were eligible for the study, if they planned to live in the village for the next 12 months. All neonates included for analysis. | **High**  All of potential confounders not considered in relation to antenatal care such as socio-economic factors, obstetric characteristics. | **Low**  Data obtained from direct measurements and used structured questionnaire developed by modifying the WHO standard for measurement of neonatal mortality | **High**  No information to indicate blinding. | **Unclear**  No reasons are given for the missing data. | **Low**  All expected outcomes (neonatal mortality) reported. |
| Engmann C, 2009 | **Low**  All participants within the 4 health districts, 12 communities participated in the study. Pregnant women were enrolled at the first prenatal visit, which was usually by 24 weeks of pregnancy between June 2005 and January 2007. | **High**  Potential confounders wealth index, area of residence, type of pregnancy etc not considered in relation to antenatal care | **Unclear**  Exposure not clearly described in the data measurement | **High**  No statement to indicate blinding and not explained in the methods part. | **Low**  No missing data. | **Low**.  All expected outcomes reported. |
| Nankabirwa,2011 | **Low**  All study participants were selected from the same Bugonkho County. Between January 2006 and September 2007, all pregnant women in the selected clusters were approached by the study team. They were eligible if they resided in the study area, were seven or more months pregnant, opted to breastfeed their infants and consented to participate in the study. Neonates of the women considered for analysis. | **High**  Important confounder are not controlled during analysis | **Low**  Used standardized tool for assessing neonatal deaths a standard (World  Health Organization (WHO) questionnaire was used to collect information) | **High**  No statement to indicate that blinding was performed | **Low**  No missing data. | **Low**  Predefined outcomes (neonatal deaths) were reported/described. |
| Kidus F, 2019 | **Low**  The cases and controls are from the same population. The study population was sample of neonates who died during the first 28 completed days after birth and sample of neonates who survived the first 28 completed  days after birth, from September 1, 2010 until  September 1, 2013. Cases were neonates (index birth) who died during the first 28 completed days after birth and controls were neonates (index birth) who survived the first 28 completed days after birth and alive during data collection. | **Low**  The major confounding variables such as educational level, pregnancy complication, income, birth interval and accessibility of facilities etc confirmed and adjusted during the analysis phase | **High**  The authors described presence of recall bias in the study. Majority of the data was three-year recall period self-reported data by mothers. Hence, this might introduced recall bias due to differential recall of information among mothers of cases and mothers of controls. | **Low**  No information to indicate that blinding was performed, but unlikely to affect outcome measurements. | **Low**  No missing data. | **Low**  All outcomes described in the results. |
| Welaga P,2013 | **Low**  All participants drawn from the same area(the Kassena-Nankana District) during the study period | **High**  Potential confounders not accounted comparing women not receiving ANC and those receiving ANC | **High**  There was recall bias particularly in the measurement of risk factors. . | **Low**  No information to indicate that blinding was performed, but unlikely to affect outcome measurements. | **Low**  Non-response rate and missing data described. No effect on the outcomes. | **Low.**  All outcomes (neonatal mortality) were included in the study description |
| Debelew GT,2014 | **Low**  All pregnant women included from the same zone(Jimma and Agaro) and their neonates followed in the same study area between September 2012 to December 2012 | **Low**  Major confounder like area of residence, educational status of the women and husband, average distance from Health facilities , Wealth quintiles, age of the mothers considered during analysis | **Low**  Data obtained by using pre-tested interviewer administered structured questionnaires which were adapted from different literatures and indicators for  neonatal care practices were adapted from the World Health  Organization (WHO) minimum neonatal care packages | **Low**  No statement to indicate that blinding was performed, but unlikely to affect outcome measurements. | **Low**  No missing data. | **Low**  All predefined neonatal outcomes were described. |
| Kolola T,2016 | **Low**  Cases and controls selected from the same area and clear definition given on it. The cases and controls group selected from comparable population. Cases were deceased new-borns within 28 days of birth while controls were infants survived | **High**  Only house hold wealth index confounders considered. Not considered all of the confounders between ANC and neonatal mortality. | **Low**  Data were collected from mothers of the cases and controls using structured questionnaires  which adopted from Ethiopian Demographic and Health Survey | **High**  The cases already known | **LOW**  No missing data. | **Low**.  All expected outcomes were described |
| Ezeh OK, 2014 | **Low**  All participants selected from the same population. All women interviewed drawn from the same population | **High**  Not considered all of potential variables which has relation with antenatal care | **Low**  A structured questionnaire was used for interviewing  the selected households for the 2008 Nigeria DHS | **Low**.  No statement to indicate blinding, but unlikely to affect outcome measurements. | **High**  There is missing data but not described how analyzed | **Low**  Neonatal mortality described clearly |
| Engmann C,2012 | **Low**  All pregnant women followed until the outcomes occur from the same area during January 2002-December 2008 | **High**  Important confounder are not controlled during analysis in relation to antenatal care. For instance , accessibility, area of residence, obstetric characteristics etc. | **Unclear**  Measurement of risk factors unclear. | **Low**  No information to indicate that blinding was performed, but unlikely to affect outcome measurements. | **Low**  No missing data. | **Low**.  All neonatal outcomes were reported. |
| Arunda M,2017 | Unclear  All participants were selected randomly at country level but didn’t show the specific areas or population. | High  Potential confounders such as accessibility, pregnancy intentions, marital status, complications etc not considered | **Low**  Risk factors clearly defined. Data were obtained from the national survey | **Low**  No information to indicate that blinding was performed, but unlikely to affect outcome measurements. | Low  No missing data. | **Low**  All expected outcomes clearly described in the study. ANC and neonatal outcomes reported in detail. |
| Orsido TT,2019 | Low  All medical records of cases and controls admitted to Wolaita Sodo University Teaching and referral hospital between October 2015 and October 2017 were included | High  All potential confounding factors related to antenatal care not considered (for instance, socio-economic status, obstetric characteristics and health facility access not considered in relation with antenatal care) . | Low  Exposure such as maternal and Neonatal related factors: ANC use, parity, gravidity, mode of delivery gestational age, birth weight, etc described clearly. | **Low**  Blinding was not described but it is absence has no effect on the outcomes. | Low  The cause of the missing data considered. (The records of 62 neonates were excluded because of predetermined exclusion criteria; 39 (3.8%) incompleteness, 15(1.5%) age above 28 days and 11(1.1%) revisits) | **Low**  The outcome described and presented clearly. |
| Farah AE,2018 | **Low**  New-borns below the age of 28 days who were admitted in the NICU from August 2014 to May 2017 were considered | **High**  The major confounding variables are not considered to eliminate possible confounders. | **Unclear**  No clear description of risk factors | **Low**  No information to indicate that blinding was performed, but unlikely to affect outcome measurements. | **Low**  Incomplete recording described but mitigation measures were taken during data collection | **Low**  The expected outcomes described clearly |
